# Draft Genome Sequence of *Streptomyces* sp. Strain IB2014011-1, Isolated from *Trichoptera* sp. Larvae of Lake Baikal

**DOI:** 10.1128/genomeA.00062-17

**Published:** 2017-04-27

**Authors:** Denis V. Axenov-Gribanov, Bogdan T. Tokovenko, Yuriy V. Rebets, Irina V. Voytsekhovskaya, Zhanna M. Shatilina, Eugenii S. Protasov, Andriy N. Luzhetskyy, Maxim A. Timofeyev

**Affiliations:** a Irkutsk State University, Institute of Biology, Irkutsk, Russia; b Baikal Research Centre, Irkutsk, Russia; c Helmholtz Center for Infection Research (HZI), Helmholtz Institute for Pharmaceutical Research Saarland (HIPS), Saarbrücken, Germany; d Universität des Saarlandes, Saarbrücken, Germany

## Abstract

Unique ecosystems with specific environmental conditions have been proven to be a promising source for isolation of new actinobacterial strains. Ancient Lake Baikal is one of the greatest examples of an ecosystem with high species biodiversity and endemicity caused by long-lasting isolated evolution and stable environmental conditions. Herein we report the draft genome sequence of *Streptomyces* sp. strain IB2014011-1, which was isolated from insect *Trichoptera* sp. larvae collected at the bottom of Lake Baikal.

## GENOME ANNOUNCEMENT

Actinobacteria are high-GC Gram-positive bacteria with high ability to produce secondary metabolites (1). It has been previously shown that actinobacteria isolated from unusual ecosystems often produce new biologically active compounds (2). Recently, we reported the isolation of new actinobacterial strains from areas of endemicity such as Lake Baikal (3) and caves (4).

Genomic DNA was extracted from *Streptomyces* sp. strain IB2014011-1, which was isolated from insect *Trichoptera* sp. larvae (3). Standard protocol was used to prepare a paired-end library. The library had reads 100 bp long, insert size 259.07 (σ = 85.71), and mean coverage 720×, as determined by postassembly mapping with bwa v 0.7.13-r1126 (mem alignment algorithm) (5). The raw sequencing data were obtained using Illumina HiSeq 2500 technology. The genome was assembled using SPAdes v 3.7 (6). A total of 73 contigs and 68 scaffolds were assembled. Scaffolding, performed using SSPACE 2.1 Premium (7), resulted in 43 scaffolds, of which 31 passed coverage and length (at least 1 kbp) thresholds. Genome annotation was performed using Prokka (8) and antiSMASH v.3 (9), followed by manual presubmission curation.

The genome of *Streptomyces* sp. IB2014011-1 is large: all scaffolds together are 8,195,763 bp long. The G+C content (71.5%) and the number of protein-coding (7,323) and tRNA (78) genes are in accordance with those of other *Streptomycetes* strains. The average gene length is 988 bp and the average coding density is 88.3%. The genome of *Streptomyces* sp. IB2014 011-1 contains at least four rRNA gene clusters, judging by the coverage of the assembled rRNA gene cluster. In addition, one ssrA transfer-messenger RNA (tmRNA) and 206 TTA codons within predicted coding sequences (CDS) were found.

Functional annotation of *Streptomyces* sp. IB2014011-1 genes within the bactNOG subset of the eggNOG v 4 database (performed using protein BLAST with an expectation value cutoff 0.001) (10) showed that 4,610 (63%) out of 7,323 protein-coding genes had at least some biological function assigned, with some of the genes assigned to more than one category. Of the remainder, 194 CDS (12.6%) had no hits against bactNOGs, and 1,857 CDS (25.4%) had hits but were not assigned to functional categories. Among the genes with functional assignment, 1,979 (27%) are implicated in metabolism, including 142 (1.9%) putatively involved in secondary metabolism.

The potential of this strain to produce secondary metabolites was analyzed with the search tool antiSMASH v.3 (9). Thirty putative secondary metabolism gene clusters were found with this method, and 55 more were predicted with the ClusterFinder algorithm.

A gene cluster of 50 is predicted to be involved in the assembly of the compound grixazone B (11) (accurate mass is 417.0616 *m/z*), which was found in the culture of the strain. All 13 g*ri* genes of the originally reported gene cluster are present, with an average nucleotide identity of 88% across the 15-kbp fragment.

Thus, the genome information provided by the draft sequence of *Streptomyces* sp. IB2014011-1 has importance for basic as well as applied microbial genomic research.

### Accession number(s).

The genome sequence of *Streptomyces* sp. IB2014011-1 has been deposited at DDBJ/ENA/GenBank under the accession number LZQS00000000. The version described in this paper is LZQS00000000.1.
